# In Vivo Transcutaneous Monitoring of Hemoglobin Derivatives Using a Red-Green-Blue Camera-Based Spectral Imaging Technique

**DOI:** 10.3390/ijms22041528

**Published:** 2021-02-03

**Authors:** Fahima Khatun, Yoshihisa Aizu, Izumi Nishidate

**Affiliations:** 1 Graduate School of Bio-Applications & Systems Engineering, Tokyo University of Agriculture and Technology, Koganei 184-8588, Japan; s186125v@st.go.tuat.ac.jp; 2 Department of Pathobiology, Faculty of Veterinary Medicine and Animal Science, Banghabandhu Sheikh Mujibur Rahman Agricultural University, Gazipur 1706, Bangladesh; 3 College of Design and Manufacturing Technology, Muroran Institute of Technology, Muroran 050-8588, Japan; aizu@mmm.muroran-it.ac.jp

**Keywords:** oxygenated hemoglobin, deoxygenated hemoglobin, methemoglobin, tissue oxygen saturation, wiener estimation method, spectral imaging, multiple regression analysis, Monte Carlo simulation

## Abstract

Cyanosis is a pathological condition that is characterized by a bluish discoloration of the skin or mucous membranes. It may result from a number of medical conditions, including disorders of the respiratory system and central nervous system, cardiovascular diseases, peripheral vascular diseases, deep vein thrombosis, and regional ischemia. Cyanosis can also be elicited from methemoglobin. Therefore, a simple, rapid, and simultaneous monitoring of changes in oxygenated hemoglobin and deoxygenated hemoglobin is useful for protective strategies against organ ischemic injury. We previously developed a red-green-blue camera-based spectral imaging method for the measurements of melanin concentration, oxygenated hemoglobin concentration (*C*_HbO_), deoxygenated hemoglobin concentration (*C*_Hb__R_), total hemoglobin concentration (*C*_HbT_) and tissue oxygen saturation (*StO*_2_) in skin tissues. We leveraged this approach in this study and extended it to the simultaneous quantifications of methemoglobin concentration (*C*_metHb_), *C*_HbO_, *C*_Hb__R_, and *StO*_2_. The aim of the study was to confirm the feasibility of the method to monitor *C*_metHb_, *C*_HbO_, *C*_Hb__R_, *C*_HbT_, and *StO*_2_. We performed in vivo experiments using rat dorsal skin during methemoglobinemia induced by the administration of sodium nitrite (NaNO_2_) and changing the fraction of inspired oxygen (FiO_2_), including normoxia, hypoxia, and anoxia. Spectral diffuse reflectance images were estimated from an RGB image by the Wiener estimation method. Multiple regression analysis based on Monte Carlo simulations of light transport was used to estimate *C*_HbO_, *C*_HbR_, *C*_metHb_, *C*_HbT_, and *StO*_2_. *C*_metHb_ rapidly increased with a half-maximum time of less than 30 min and reached maximal values nearly 60 min after the administration of NaNO_2_, whereas *StO*_2_ dramatically dropped after the administration of NaNO_2_, indicating the temporary production of methemoglobin and severe hypoxemia during methemoglobinemia. Time courses of *C*_HbT_ and *StO*_2_, while changing the FiO_2_, coincided with well-known physiological responses to hyperoxia, normoxia, and hypoxia. The results indicated the potential of this method to evaluate changes in skin hemodynamics due to loss of tissue viability and vitality.

## 1. Introduction

**Figure 1:**
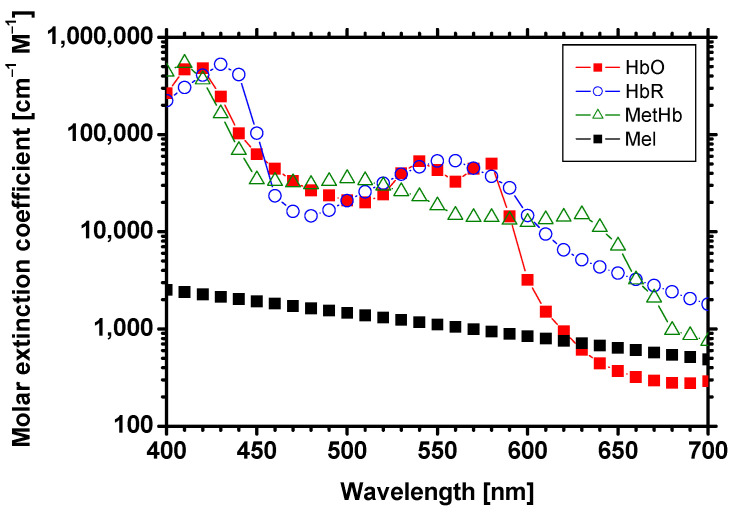
Extinction coefficient spectra of typical hemoglobin derivatives (oxygenated hemoglobin HbO^10^, deoxygenated hemoglobin HbR^10^, and methemoglobin^11^) and melanin^12^ in visible wavelength region between 400 and 700 nm.

A patient with a bluish discoloration in the skin, known as cyanosis, is alarming to a medical doctor. Such cases may be severely hypoxemic or hypoxic, constituting a medical emergency [1]. Cyanosis first appears in the extremities when oxygen demand outstrips the supply, resulting in a high amount of deoxygenated blood accumulating in blood-perfused dermal tissues [2,3]. This may result from a number of medical conditions, including reduced cardiac output (heart failure), peripheral vasoconstriction (hypothermia), and regional ischemia (arterial thrombosis) [1,4,5]. Cyanosis can also be elicited from dysfunctional forms of hemoglobin, such as methemoglobin (metHb) [2,6,7]. Methemoglobin is an abnormal form of hemoglobin that oxidizes iron molecules within hemoglobin [6,8,9]. Figure 1 shows the extinction coefficient spectra of typical hemoglobin derivatives and melanin at visible wavelengths between 400 and 700 nm [10,11,12].

The absorption spectrum of hemoglobin depends primarily on whether or not the blood is oxygenated. Thus, there are two main derivatives, i.e., oxyhemoglobin in oxygenated blood and deoxyhemoglobin in deoxygenated blood. There are also hemoglobin derivatives that are incapable of transporting oxygen molecules. These are collectively known as dyshemoglobins, which include methemoglobin, carboxyhemoglobin, and sulfhemoglobin. The absorption can differ drastically between dysfunctional hemoglobin and functional hemoglobin [13,14]. Methemoglobin is unable to bind oxygen, so if a large amount of an individual’s hemoglobin is converted to methemoglobin, tissue oxygenation is impaired. Furthermore, increased fractions of methemoglobin shift the oxygen dissociation curve to the left, which further impairs tissue oxygenation [15,16]. Given the low partial pressure of oxygen, hypoxia ensues [17,18]. In a normal human subject, less than 1–2% methemoglobin is present [6]. An abnormal level of methemoglobin (methemoglobinemia) may result from congenital enzyme deficiencies [9,19] or exposure to certain chemicals and medications turning hemoglobin into a dysfunctional form of hemoglobin [20,21,22,23,24]. These chemical agents include drugs used in hospitals and health care settings, such as some local anesthetics (e.g., benzocaine and lidocaine [20,21,22]) and antibiotics (e.g., dapsone [23]), as well as nitrates and nitrites present in fertilizers that may contaminate food and water supplies [24,25]. The initial symptoms appear as a bluish discoloration of the skin when the methemoglobin level is at least 10–20% of the total hemoglobin [6,7,8,9,10,11,12,13,14,15,16,17,18,19,20,21,22,23,24,25,26]. This is potentially life threatening and a high mortality rate is often observed when the methemoglobin percentage exceeds 70% [6,7,8,9,10,11,12,13,14,15,16,17,18,19,20,21,22,23,24,25,26]. While, in many cases, methemoglobinemia is reversible after treating with methylene blue [27], some unusual cases do not respond to this substance [28]. Therefore, the evaluation of methemoglobin concentration is important for clinical applications. Furthermore, information on the spatial distribution of methemoglobin would aid in the diagnosis and treatment of diseases. The fast measurement of methemoglobin is very important to surgeons when anesthetics are applied, allowing them to react quickly by revealing the concentration change following the treatment. Moreover, it provides information for differential diagnoses in patients with mechanical airway obstruction or pulmonary embolus [29], and in infants with congenital cyanosis or sepsis [9].

Current devices, such as co-oximeters and blood gas analyzers, are frequently applied to evaluate the abnormal presence of methemoglobin in blood samples [30]. The readings provided by co-oximeters often provide false positives in the presence of sulfhemoglobin and methylene blue [31,32]. The arterial blood gas analyzer only measures dissolved oxygen, and not the actual amount of oxygen bound to hemoglobin in the blood. This also results in a false positive diagnosis for methemoglobinemia even though the metHb concentration is high [9,31]. In addition to these two methods, other techniques, such as broadband optical spectroscopy [33,34,35,36] and photoacoustic imaging [37,38] have the potential for the non-invasive in vivo monitoring of methemoglobin absorption properties.

Diffuse reflectance spectroscopy (DRS) has been utilized for the evaluation of skin chromophores (melanin, oxygenated hemoglobin and deoxygenated hemoglobin) [39,40,41] and tissue oxygen saturation [39,41,42]. Multi-spectral imaging, based on DRS, has been extensively researched for evaluating the spatial distribution of chromophore content in living tissue [43,44,45]. On the other hand, the reconstruction of multispectral images from a red/green/blue (RGB) image acquired by a digital RGB camera is a promising method for performing rapid and cost-effective multispectral imaging. Several reconstruction techniques for multispectral images, such as the pseudo-inverse method [46,47,48,49], finite-dimensional modeling [48,50], nonlinear estimation method [51], and Wiener estimation method (WEM) [52,53,54,55] have been studied. Among these reconstruction techniques, the WEM is one of the most promising methods for the establishment of a compact and affordable imaging system that can simultaneously evaluate the spatiotemporal distributions of chromophores in clinical point-of-care testing due to its simplicity, cost-effectiveness, accuracy, time efficiency, and possibility of high-resolution image acquisition. Such a device would have an enormous impact on the health of people of all ages worldwide. 

In the existing literature, a spectral imaging method based on the WEM has been proposed for the measurements of melanin, oxygenated hemoglobin, and deoxygenated hemoglobin in skin tissues. The WEM has also been applied to the visualization of spatiotemporal changes in peripheral hemodynamics in response to physiological stimuli [56]. In this approach, multiple regression analysis is performed by using the spectral image reconstructed by the WEM at wavelengths between 500 and 600 nm as a response variable and the known extinction coefficient spectra of melanin, oxygenated hemoglobin, and deoxygenated hemoglobin as predictor variables to provide multiple regression coefficients. The concentrations of melanin and hemoglobin are then determined from the regression coefficients using empirical formulae that are deduced numerically in advance. A Monte Carlo simulation (MCS) of light transport in a human skin model is carried out to numerically establish the empirical formulae. We leveraged this approach in our study and extended it to the simultaneous quantification of methemoglobin, oxygenated hemoglobin, and deoxygenated hemoglobin. Multispectral diffuse reflectance images of in vivo skin are estimated from an RGB image captured by a digital RGB camera. An MCS-based multiple regression analysis for the estimated absorbance spectra at 16 wavelengths (500 to 650 nm at 10 nm intervals) is then used to quantify the chromophore concentrations. In order to confirm the feasibility of this method to evaluate chromophore concentrations, we performed in vivo experiments using rat dorsal skin during methemoglobinemia induced by the administration of sodium nitrite (NaNO_2_) and changing the fraction of inspired oxygen (FiO_2_), including normoxia, hypoxia, and anoxia.

## 2. Results

**Figure 2:**
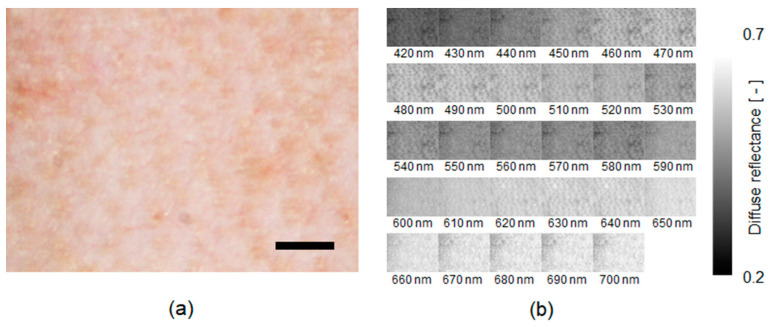
Typical results obtained from in vivo rat dorsal skin under normal conditions: (**a**) raw RGB image and (**b**) estimated images of spectral diffuse reflectance by Wiener estimation. A scale bar (black line in the RGB image) indicates 1.0 mm.

**Figure 3:**
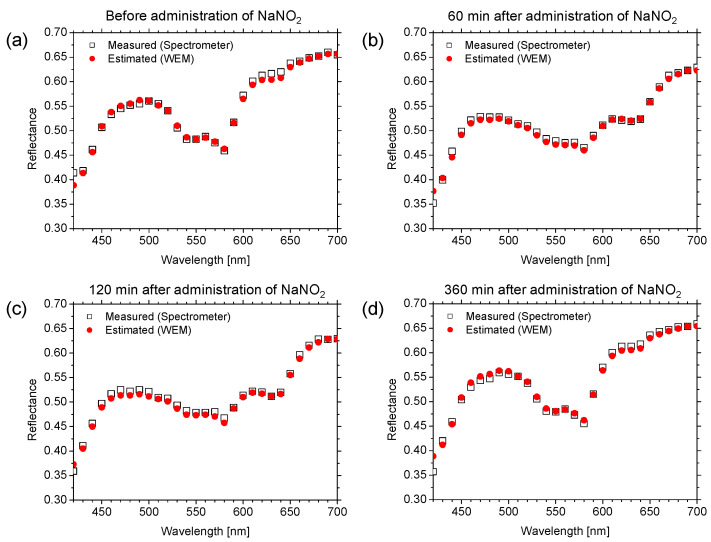
Comparisons of estimated reflectance spectra averaged over entire region of spectral images and reference spectra measured by spectrometer: (**a**) before administration of NaNO_2_, (**b**) 60 min after administration of NaNO_2_, (**c**) 120 min after administration of NaNO_2_, and (**d**) 360 min after administration of NaNO_2_.

Figure 2 shows typical spectral reflectance images estimated from the RGB image of in vivo rat dorsal skin under normal conditions by the WEM. Spectral reflectance images of the skin were successfully reconstructed from the RGB image using the proposed method. Figure 3 shows the reflectance spectra estimated using the WEM and the reflectance spectra measured by the spectrometer (a) before the administration of NaNO_2_, (b) 60 min after the administration of NaNO_2_, (c) 120 min after the administration of NaNO_2_, and (d) 360 min after the administration of NaNO_2_. The reflectance spectra estimated using the WEM are comparable to the spectra measured by the spectrometer for the different time points. The estimated spectra after the administration of NaNO_2_ are dominated by the spectral characteristics of methemoglobin. The values of the goodness-of-fit coefficient (GFC) obtained from four rats after the administration of NaNO_2_ summarized in Table 1 indicate an accurate spectral reconstruction by the WEM.

**Figure 4:**
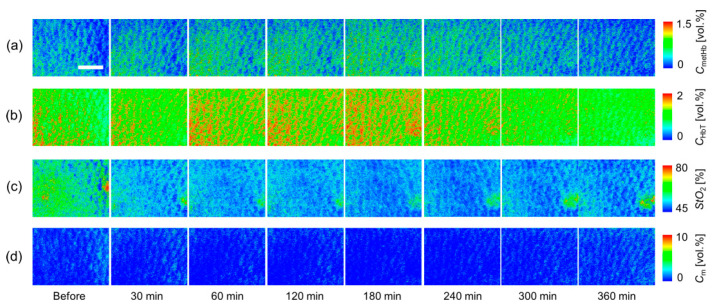
Typical sequential images of (**a**) *C*_metHb_, (**b**) *C*_HbT_, (**c**) *StO*_2_, and (**d**) *C*_m_ obtained from rat dorsal skin before and after the administration of NaNO_2_ at dose of 50 mg/kg. A scale bar (white line in the *C*_metHb_ image for before) indicates 2.0 mm.

**Figure 5:**
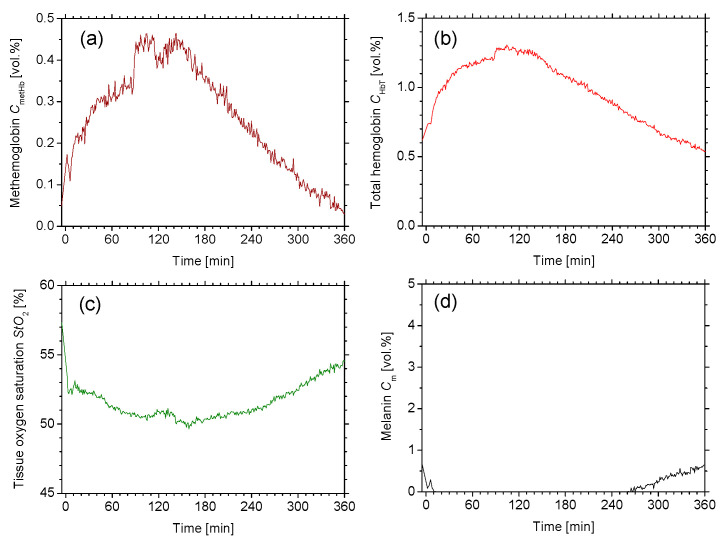
Typical time courses of (**a**) *C*_metHb_, (**b**) *C*_HbT_, (**c**) *StO*_2_, and (**d**) *C*_m_ averaged over entire region of each estimated image.

Figure 4 shows typical sequential images of (a) *C*_metHb_, (b) *C*_HbT_, (c) *StO*_2_, and (d) *C*_m_ obtained from rat dorsal skin before and after the administration of NaNO_2_ at a dose of 50 mg/kg. Figure 5 shows the typical time courses of (a) Δ*C*_metHb_, (b) Δ*C*_HbT_, (c) Δ*StO*_2_, and (d) Δ*C*_m_ averaged over the entire region of each estimated image. *C*_metHb_ increased after the administration of NaNO_2_ and returned to normal levels at 360 min after the administration of NaNO_2_. The value of *StO*_2_ decreased after the administration of NaNO_2_, indicating temporary hypoxemia caused by methemoglobinemia. There was a profound increase in *C*_Hb__T_ after the administration of NaNO_2_. In spite of the remarkable changes in *C*_metHb_, *C*_Hb__T_ and *StO*_2_, *C*_m_ remained almost unchanged during the measurements.

**Figure 6:**
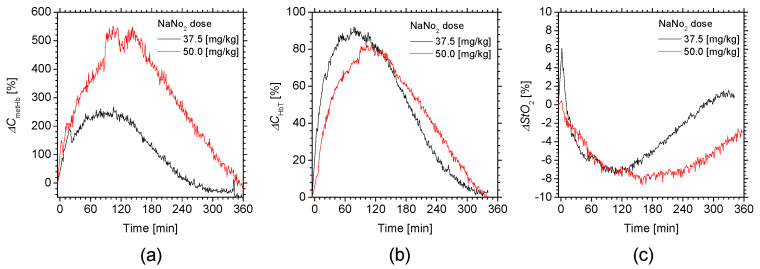
Time courses of (**a**) Δ*C*_metHb_, (**b**) Δ*C*_HbT_, and (**c**) Δ*StO*_2_ Δ*C*_m_ averaged over two rats for different doses of NaNO_2_.

Figure 6 shows the time courses of (a) Δ*C*_metHb_, (b) Δ*C*_HbT_, and (c) Δ*StO*_2_ averaged over two rats for different doses of NaNO_2_. The values of Δ*C*_metHb_ rapidly increased with a half-maximum time of less than 30 min, and the time required to return to the normal levels increased proportionally with the dose. On the other hand, for each dose condition, the time course of Δ*StO*_2_ dramatically dropped at 120 min after the administration of NaNO_2_, and then gradually increased again. The values of Δ*StO*_2_ were negatively correlated to those of Δ*C*_metHb_. 

**Figure 7:**
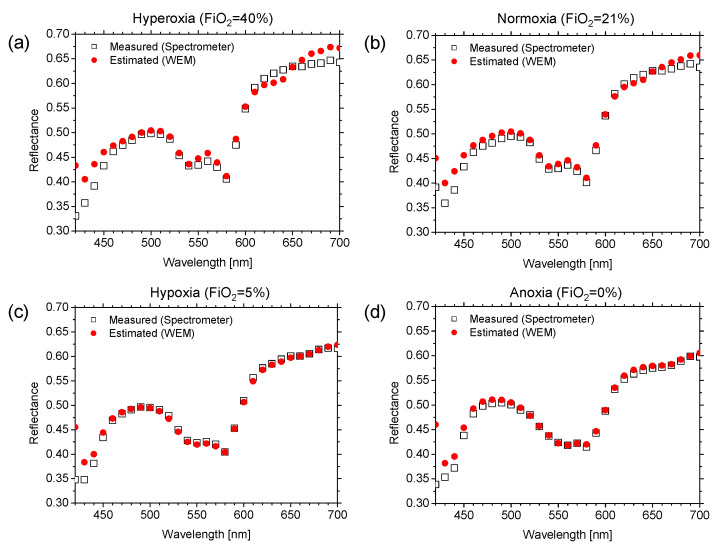
Comparisons of estimated reflectance spectra and reference spectra measured by spectrometer for (**a**) hyperoxia (FiO_2_ = 40%), (**b**) normoxia (FiO_2_ = 21%), (**c**) hypoxia (FiO_2_ = 5%), and (**d**) anoxia (FiO_2_ = 0%).

Figure 7 shows the reflectance spectra estimated using the WEM and the reflectance spectra measured by the spectrometer for (a) hyperoxia (FiO_2_ = 40%), (b) normoxia (FiO_2_ = 21%), (c) hypoxia (FiO_2_ = 5%), and (d) anoxia (FiO_2_ = 0%). The reflectance spectra estimated using the WEM are comparable to the spectra measured by the spectrometer for the different time points. The estimated spectra at hyperoxia and hypoxia are dominated by the spectral characteristics of oxygenated hemoglobin and deoxygenated hemoglobin, respectively. The values of GFC obtained from three rats while changing FiO_2_ are summarized in Table 2, which indicates a successful spectral reconstruction by the WEM.

**Figure 8:**
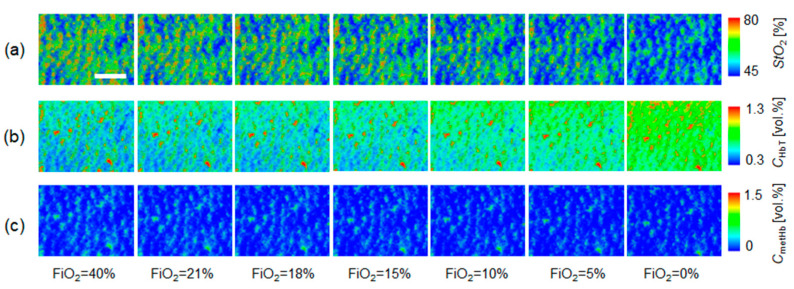
Typical sequential images of (**a**) *StO*_2_, (**b**) *C*_HbT_, and (**c**) *C*_metHb_ obtained from rat dorsal skin during changes in FiO_2_. A scale bar (white line in the *StO*_2_ image for FiO_2_ = 40%) indicates 2.0 mm.

**Figure 9:**
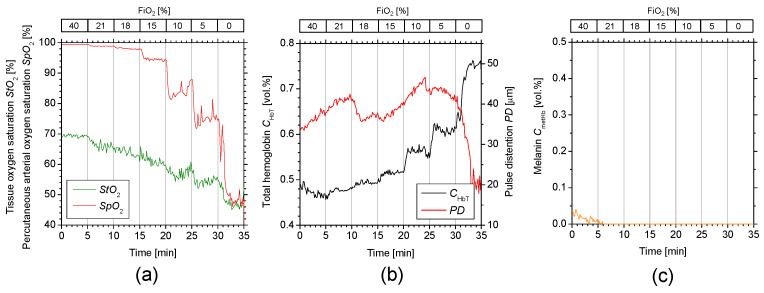
Typical time courses of (**a**) *StO*_2_, (**b**) *C*_HbT_, and (**c**) *C*_metHb_ averaged over entire region of each corresponding image shown in Figure 8. Time courses of *SpO*_2_ and *PD* were also compared with *StO*_2_ and *C*_HbT_, respectively.

Figure 8 shows typical sequential images of *C*_HbT_, *StO*_2_, and *C*_metHb_ obtained from rat dorsal skin during changes in FiO_2_. Figure 9 shows the time courses of (a) *C*_HbT_, (b) *StO*_2_, and (c) *C*_metHb_ averaged over the entire region of each image shown in Figure 8 during changes in FiO_2_. Time courses of percutaneous oxygen saturation (*SpO*_2_) and pulse distention (*PD*) are also compared with *StO*_2_ and *C*_HbT_, respectively, in Figure 9. The value of *SpO*_2_ dropped remarkably when FiO_2_ was below 15%. Values of *StO*_2_ and *C*_HbT_ gradually decreased and increased, respectively, according to reductions in FiO_2_. The value of *PD* gradually increased after the onset of hypoxia. In spite of the remarkable changes in *C*_HbT_ and *StO*_2_, there was no significant change in *C*_metHb_ during the measurements.

**Figure 10:**
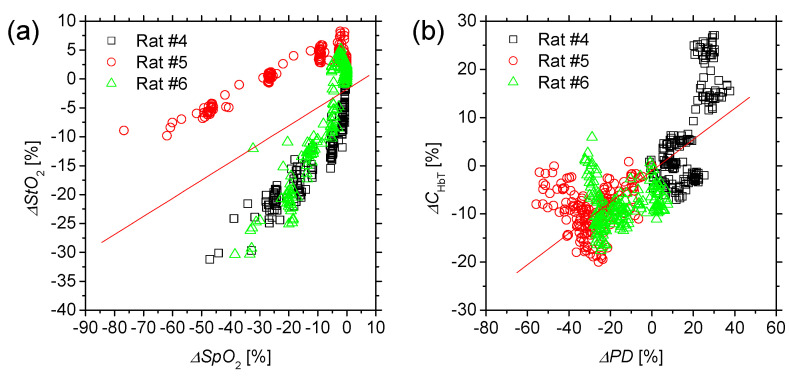
Scatter plots of (**a**) Δ*SpO*_2_ vs. Δ*StO*_2_ and (**b**) Δ*C*_HbT_ vs. Δ*PD* obtained from three rats.

Figure 10 shows scatter plots of (a) Δ*SpO*_2_ vs. Δ*StO*_2_ and (b) Δ*C*_HbT_ vs. Δ*PD* obtained from the three rats. As can be seen, the values of Δ*StO*_2_ and Δ*C*_HbT_ correlated with those of *SpO*_2_ and Δ*PD*, respectively. The correlation coefficient between Δ*SpO*_2_ and Δ*StO*_2_ and that between Δ*C*_HbT_ and Δ*PD* were *R* = 0.48 (*p* < 0.0001) and *R* = 0.73 (*p* < 0.0001), indicating that *StO*_2_ and *C*_HbT_ were moderately correlated with *SpO*_2_ and *PD*, respectively.

## 3. Discussion

In the present study, a model of light transport in skin tissue is applied to analyze the reflectance spectra of rat dorsal skin during methemoglobinemia induced by the administration of NaNO_2_ and changing the FiO_2_ using an RGB imaging instrument. With the RGB imaging technique, it was possible to obtain images of oxygenated hemoglobin concentration, deoxygenated hemoglobin concentration, total hemoglobin concentration, and tissue oxygen saturation. The WEM was introduced to reconstruct spectral diffuse reflectance images from an RGB image. The accuracy in spectral reconstruction was validated by comparing the estimated spectra by the WEM with the measured spectra by spectrometer. The results showed that the WEM can present an accurate spectral reconstruction with the average GFC more than 0.998 (Table 1 and Table 2). Considering noise in the camera response for **W** or using higher order terms of elements in vector **v** may improve the accuracy of the WEM [53,54]. In this study, we used diffuse reflectance spectra obtained from the skin of albino rats which contained no melanin granules, to establish the matrix **W** for the WEM. Therefore, we did not confirm the feasibility of the proposed method to evaluate the change in melanin level. When the proposed method is applied to human subjects, the Wiener estimation matrix should be established with diffuse reflectance spectra obtained from the skin of human subjects with different skin phototypes or skin colors.

We examined the hemodynamic responses of rat dorsal skin after the administration of NaNO_2_. We observed the profound increase in *C*_metHb_ after the administration of NaNO_2_ and the subsequent decrease (Figure 4a and Figure 5a). The time course of *C*_metHb_ was indicative of temporary methemoglobinemia. We also observed that the value of *C*_metHb_ rapidly increased with a half-maximum time of less than 30 min, and the time required to return to the normal levels increased proportionally with the dose (Figure 6a). The dose-dependent change in *C*_metHb_ is compatible with previously reported results [57]. The value of *StO*_2_ dramatically dropped at 60 min after the administration of NaNO_2_, indicating temporary hypoxemia caused by methemoglobinemia, as expected (Figure 4c and Figure 5c). The time required to return to the normal *StO*_2_ levels increased proportionally with the dose (Figure 6c). The results for *StO*_2_ indicated that the proposed method can be used to evaluate peripheral hemoglobin oxygenation level during methemoglobinemia. The value of *C*_HbT_ was remarkably increased after the administration of NaNO_2_ (Figure 4b, Figure 5b and Figure 6b). The profound increase in *C*_HbT_ indicated an elevated cardiac output caused by tachycardia during methemoglobinemia [58] and the resultant increase in peripheral blood volume to compensate for the hypoxia and hypoxemia.

We also examined the hemodynamic responses of rat dorsal skin while changing FiO_2_. We demonstrated that values of *StO*_2_ and *C*_HbT_ obtained by the proposed method significantly decreased and increased, respectively, as FiO_2_ decreased (Figure 8 and Figure 9). Under the normoxia (FiO_2_ = 21%), the average value of 72.6 ± 4.6% for *StO*_2_ obtained by our method is lower than the average value of 98.8 ± 0.3% for *SpO*_2_ measured by a pulse oximeter. The value of *StO*_2_ estimated by the proposed method represents the oxygen saturation for the mixture of arterio-venous blood. Almost 75% of the total blood volume in the whole body is contained within the veins and venules, whereas 25% of it is contained within the arteries and arterioles. Assuming that the blood volume ratio of venules and arterioles in the skin tissue is similar to that of the whole body, and the values of arterial oxygen saturation *SaO*_2_ and venous oxygen saturation *SvO*_2_ under the normoxic condition are 98% and 75%, respectively, the tissue oxygen saturation of skin is calculated to be 80.6%. This value is close to the average value of 72.6 ± 4.6% for *StO*_2_, obtained by our method. We observed that the value of *StO*_2_ decreased gradually as FiO_2_ decreased, whereas *SpO*_2_ decreased dramatically when FiO_2_ was below 15% (Figure 9a). Those changes in both *SpO*_2_ and *StO*_2_ are due to hypoxemia, which is due to hypoxia. We also observed the profound increase in *PD* when FiO_2_ is below 18% (Figure 9b). The increase in *PD* is probably due to an elevated cardiac output caused by tachycardia and the resultant increase in peripheral blood volume to compensate for the hypoxia and hypoxemia; this was in agreement with the time course of *C*_HbT_ obtained by the proposed method. The time courses of *C*_HbT_ and *StO*_2_ during changes in FiO_2_ were consistent with well-known physiological responses to changes in FiO_2_.

We note that the accuracy of the quantification of each chromophore also depends on the actual thicknesses of the epidermis and the dermis and the light scattering properties of the target skin tissue. In the present study, we assumed the typical thicknesses of the epidermis and the dermis in the MCS to derive the empirical model. The assumed thicknesses of epidermis and the dermis could have an impact on the results of chromophore concentrations. The probability that light is absorbed by melanin in the epidermis will be higher than the probability that light is absorbed by hemoglobin in the dermis as the epidermis becomes thicker. Therefore, if the epidermal thickness is larger than the assumed typical value, melanin in epidermis or hemoglobin derivatives could be under- or overestimated, respectively. The current empirical formulae for chromophores were derived from the MCS with a typical reduced scattering coefficient spectrum derived from the literature data. However, the scattering spectrum usually differs among body parts, and may vary with the age of the subjects. The correct estimation of the scattering properties or consideration of the variations in the scattering spectrum is essential to precisely estimate the chromophore concentrations.

The present method lacks depth resolution, because it relies on the integration of all diffuse reflection information along the depth direction. Moreover, the present method is based on the MCS model, which assumes uniformly distributed scattering and absorption properties. The current algorithm performs multiple regression analysis using each pixel in the spectral image to calculate the multiple regression coefficients. This process is relatively time-consuming, and the computational load increases when the number of pixels in an image for analysis increases. Therefore, this method may not be suitable for real-time high temporal and spatial resolution imaging of chromophores in vivo. The current empirical formulae for chromophores were derived from the MCS with a typical reduced scattering coefficient spectrum. However, the reduced scattering coefficient spectrum usually differs among body parts and may vary with the age of the subjects. Correct estimation of the scattering properties or consideration of the variations in the reduced scattering coefficient spectrum is essential to precisely estimate the chromophore concentrations.

## 4. Materials and Methods

### 4.1. Reconstruction of Spectral Image by Wiener Estimation Method 

The response of a digital color camera with spatial coordinates (*x*, *y*) with an *i*th (*i* = 1, 2, 3) color channel, or Red, Green, and Blue, can be calculated as:

(1) $$v_{i}(x,\;y)=\int u_{i}\left(\lambda \right)E\left(\lambda \right)S\left(\lambda \right)r\left(x,y;\lambda \right)d\lambda ,$$

where *λ* is the wavelength, *u_i_*(*λ*) is the transmittance spectrum of the *i*th filter, *E*(*λ*) is the spectrum of the illuminant, *S*(*λ*) is the sensitivity of the camera, and *r* (*x*, *y*; *λ*) is the reflectance spectrum in the spatial coordinates (*x*, *y*). For convenience, Equation (1) is expressed in discrete vector notation as:

(2) v=Fr,

where **v** is a vector with a three-element column and **r** is a vector with a *k* element column, which corresponds to the reflectance spectrum of a pixel of an image. **F** is a 3 × *k* matrix and is expressed as:

(3) F=UES,

where **U** = [u_1_, u_2_, u_3_]*^T^*. Column vector *u_i_* denotes the transmittance spectrum of the *i*th filter and [ ]*^T^* represents the transposition of a vector. **E** and **S** are *k* × *k* diagonal matrices and represent the spectrum of the illuminant and the sensitivity of the camera, respectively. In this study, we use the known spectral profiles of the illuminant and camera published by the manufacturers. The Wiener estimation of **r** is given by:

(4) $$\tilde{r}=Wv,$$

where **W** is the Wiener estimation matrix. The purpose of **W** is to minimize the least squares error between the original and estimated reflectance spectra. In this case, the least squares error is expressed as:

(5) $$e=\langle {\left(r-\tilde{r}\right)}^{t}\left(r-\tilde{r}\right)\rangle ,$$

From Equations (4) and (5), the least squares error is rewritten as:

(6) $$e=\langle {\left(r-\tilde{r}\right)}^{t}\left(r-\tilde{r}\right)\rangle =\langle r^{t}r\rangle -W\langle r^{t}v\rangle -W^{t}\langle v^{t}r\rangle +W^{t}W\langle v^{t}v\rangle ,$$

The minimization of the least squares error requires that the partial derivative of *e* with respect to **W** is zero as:

(7) $$\frac{\partial e}{\partial W}=-\langle r^{t}v\rangle +W^{t}\langle v^{t}v\rangle =0\;,$$

From Equation (7), the matrix W is derived as:

(8) $$W=\langle rv^{T}\rangle {\langle vv^{T}\rangle }^{-1}=\langle rv^{T}\rangle F^{T}{\left(F\langle rr^{T}\rangle F^{T}\right)}^{-1},$$

where 〈〉 is an ensemble-averaging operator. To derive the matrix **W**, the autocorrelation matrix $\langle rr^{T}\rangle$ is required. In this study, we determined $\langle rr^{T}\rangle$ on the basis of 440 different reflectance spectra obtained from rat dorsal skin under various physiological conditions. Discrete reflectance values ranging from 420 to 700 nm at 10 nm intervals were extracted from the raw reflectance spectrum measured by the spectrometer and assigned to the 29 elements of vector r. A matrix $rr^{T}$ with 29 × 29 elements was derived by multiplying the vector r and its transposition vector $r^{T}$. This procedure was applied to each reflectance sample and 440 different matrices of $rr^{T}$ were derived. The autocorrelation matrix $\langle rr^{T}\rangle$ was calculated by averaging the corresponding elements over all samples.

### 4.2. Estimation of Chromophores Based on Multiple Regression Analysis 

We modified the approach proposed by Nishidate et al. [56], which was designed to quantify the three major chromophores of oxygenated hemoglobin, deoxygenated hemoglobin, and melanin. In this study, we considered the four chromophores of methemoglobin, oxygenated hemoglobin, deoxygenated hemoglobin, and melanin in the skin tissue model. The absorbance spectrum *A*(*λ*) was defined as

(9) $$A\left(\lambda \right)=\log_{10}\frac{1}{r\left(\lambda \right)}$$

where *r*(*λ*) is the diffuse reflectance spectrum normalized by the incident light spectrum. Because attenuation due to light scattering can be treated as a pseudochromophore [59,60], the attenuation spectrum *A*(*λ*) can be approximated as the sum of attenuations due to absorption and scattering in the skin tissue, as follows:

(10) $$\begin{array}{ll} A\left(\lambda \right) & =C_{\mathrm{metHb}}l_{d}\left(\lambda ,C_{\mathrm{metHb}},C_{\mathrm{HbO}},C_{\mathrm{HbR}},\mu_{s}{}^{\prime }\right)\varepsilon_{\mathrm{metHb}}\left(\lambda \right)\\ & +C_{\mathrm{HbO}}l_{d}\left(\lambda ,C_{\mathrm{metHb}},C_{\mathrm{HbO}},C_{\mathrm{HbR}},\mu_{s}{}^{\prime }\right)\varepsilon_{\mathrm{HbO}}\left(\lambda \right)\\ & +C_{\mathrm{HbR}}l_{d}\left(\lambda ,C_{\mathrm{metHb}},C_{\mathrm{HbO}},C_{\mathrm{HbR}},\mu_{s}{}^{\prime }\right)\varepsilon_{\mathrm{HbR}}\left(\lambda \right)\\ & +C_{m}l_{e}\left(\lambda ,C_{m},\mu_{s}{}^{\prime }\right)\varepsilon_{m}\left(\lambda \right)+S\left(\lambda ,\mu_{s}{}^{\prime }\right) \end{array}$$

where *C* is the concentration, *l* is the mean path length, *ε*(*λ*) is the extinction coefficient, and *S*(*λ*, *μ_s_’*) indicates attenuation due to light scattering in the tissue. The subscripts metHb, HbO, HbR, and m indicate methemoglobin, oxygenated hemoglobin, deoxygenated hemoglobin, and melanin, respectively. By using *A*(*λ*) at *λ* = 500 to 650 nm at 10 nm intervals, as the response variable and *ε*(*λ*) in the same wavelength range as the predictor variables, the multiple regression analysis can be performed as

(11) $$A\left(\lambda \right)=a_{\mathrm{metHb}}\varepsilon_{\mathrm{metHb}}\left(\lambda \right)+a_{\mathrm{HbO}}\varepsilon_{\mathrm{HbO}}\left(\lambda \right)+a_{\mathrm{HbR}}\varepsilon_{\mathrm{HbR}}\left(\lambda \right)+a_{m}\varepsilon_{m}\left(\lambda \right)+a_{0}$$

where *a*_metHb_, *a*_HbO_, *a*_HbR_, *a*_m_, and *a*_0_ are the regression coefficients. We refer to this multiple regression analysis as MRA1. The multiple regression coefficients *a*_metHb_, *a*_HbO_, *a*_HbR_, and *a*_m_ describe the degree of contribution of *ε*_metHb_(*λ*), *ε*_HbO_(*λ*), *ε*_HbR_(*λ*), and *ε*_m_(*λ*), respectively, to *A*(*λ*). Therefore, the values of *a*_metHb_, *a*_HbO_, *a*_HbR_, and *a*_m_ are closely related to *C*_metHb_, *C*_HbO_, *C*_HbR_, and *C*_m_, respectively. We used the multiple regression coefficients *a*_metHb_, *a*_HbO_, *a*_HbR_, *a*_m,_ and *a*_0_ to estimate the concentrations of *C*_metHb_, *C*_HbO_, *C*_HbR_, and *C*_m_. For this purpose, we assumed the empirical formulae for *C*_metHb_, *C*_HbO_, *C*_HbR_, and *C*_m_ as

(12) $$C_{\mathrm{metHb}}=b_{metHb}\cdot a$$

(13) $$C_{\mathrm{HbO}}=b_{HbO}\cdot a$$

(14) $$C_{\mathrm{HbR}}=b_{HbR}\cdot a$$

(15) $$C_{m}=b_{m}\cdot a$$

where

(16) $$a={\left[1,\;a_{\mathrm{metHb}},\;a_{\mathrm{HbO}},\;a_{\mathrm{HbR}},\;a_{m},\;a_{0},\cdots higher\;order\;terms\cdots \right]}^{T}$$

(17) $$b_{metHb}=\left[b_{\mathrm{metHb},0},\;b_{\mathrm{metHb},1},\;b_{\mathrm{metHb},2},\cdots ,b_{\mathrm{metHb},z-1}\right]$$

(18) $$b_{HbO}=\left[b_{\mathrm{HbO},0},\;b_{\mathrm{HbO},1},\;b_{\mathrm{HbO},2},\cdots ,b_{\mathrm{HbO},z-1}\right]$$

(19) $$b_{HbR}=\left[b_{\mathrm{HbR},0},\;b_{\mathrm{HbR},1},\;b_{\mathrm{HbR},2},\cdots ,b_{\mathrm{HbR},z-1}\right]$$

(20) $$b_{m}=\left[b_{m,0},\;b_{m,1},\;b_{m,2},\cdots ,b_{m,z-1}\right]$$

The symbol [ ]^T^ represents the transposition of a vector. The coefficient vectors **b**_metHb_, **b**_HbO_, **b**_HbR_, and **b**_m_ are unknown and must be determined before estimating *C*_metHb_, *C*_HbO_, *C*_HbR_, and *C*_m_. The number of components in vectors **a**, **b**_metHb_, **b**_HbO_, **b**_HbR_, and **b**_m_ is assumed to be *z*. We conducted further multiple regression analyses to establish the vectors **b**_metHb_, **b**_HbO_, **b**_HbR_, and **b**_m_. In this second multiple regression analysis, *C*_metHb_, *C*_HbO_, *C*_HbR_, and *C*_m_ were regarded as dependent variables, and the five regression coefficients (*a*_metHb_, *a*_HbO_, *a*_HbR_, *a*_m_, and *a*_0_) that were obtained from MRA1 were regarded as independent variables to determine the regression equations for *C*_metHb_, *C*_HbO_, *C*_HbR_, and *C*_m_. We refer to this analysis as MRA2.

We simulated the diffuse reflectance spectra of skin tissues by using the MCS for light transport^38^ to derive the datasets of chromophore concentrations and the multiple regression coefficients *a*_metHb_, *a*_HbO_, *a*_HbR_, *a*_m_, and *a*_0_. The simulation model consisted of two layers representing the epidermis and dermis. In total, 5,000,000 photon packets were launched in a single simulation of diffuse reflectance at each wavelength. The absorption coefficients for oxygenated hemoglobin *μ_a_*_,HbO_(*λ*), deoxygenated hemoglobin *μ_a_*_,HbR_(*λ*), methemoglobin *μ_a_*_,metHb_(*λ*), and melanin *μ_a_*_,m_(*λ*) were obtained from the values of *ε*_HbO_(*λ*), *ε*_HbR_(*λ*), *ε*_metHb_(*λ*), and *ε*_mel_(*λ*), shown in Figure 1. The absorption coefficients for the epidermis for 10 different melanin concentrations were input for the epidermis layer in the MCS as *C*_m_ = 1 to 10 vol.% at 1 vol.% intervals. We assumed that whole blood with 150 g/L of hemoglobin is 100% volume concentration of total hemoglobin (*C*_HbT_ = 100 vol.%). The sum of the absorption coefficients for oxygenated hemoglobin *μ_a_*_,HbO_(*λ*), deoxygenated hemoglobin *μ_a_*_,HbR_(*λ*), and methemoglobin *μ_a_*_,metHb_(*λ*) represents the absorption coefficients for total hemoglobin *μ_a_*_,HbT_(*λ*). The absorption coefficients for total hemoglobin *μ_a_*_,HbT_(*λ*) for the values of *C*_HbT_ = 0.2 to 1.0 vol.% at 0.2 vol.% intervals were input for the dermis layer in the MCS. Tissue oxygen saturation (*StO*_2_) was determined by *μ_a_*_,HbO_(*λ*)/*μ_a_*_,HbT_(*λ*) and values ranging from 0% to 100% were used for the simulation. The refractive index for the epidermis and dermis layers was assumed to be the same and was fixed at 1.4. The thicknesses of the epidermis and dermis layers were set to 0.06 and 4.94 mm, respectively. The reduced scattering coefficient *μ_s_’*(*λ*) calculated from typical values for the scattering coefficient *μ_s_*(*λ*) [41] and anisotropy factor *g*(*λ*) [41] were used for both the epidermis and dermis layers. In total, 1550 diffuse reflectance spectra at *λ* = 500 to 650 nm at 10 nm intervals were simulated under the various combinations of *C*_metHb_, *C*_HbO_, *C*_HbR_, and *C*_m_. The MRA1 analysis for each simulated spectrum generated 1550 sets of vector **a** and concentrations of *C*_metHb_, *C*_HbO_, *C*_HbR_, and *C*_m_. The coefficient vectors **b**_metHb_, **b**_HbO_, **b**_HbR_, and **b**_m_ were determined statistically by performing MRA2. Once **b**_metHb_, **b**_HbO_, **b**_HbR_, and **b**_m_ were obtained, *C*_metHb_, *C*_HbO_, *C*_HbR_, and *C*_m_ were calculated from *a*_metHb_, *a*_HbO_, *a*_HbR_, *a*_m_, and *a*_0_, which were derived from MRA1 for the measured reflectance spectrum. 

### 4.3. Imaging System 

**Figure 11:**
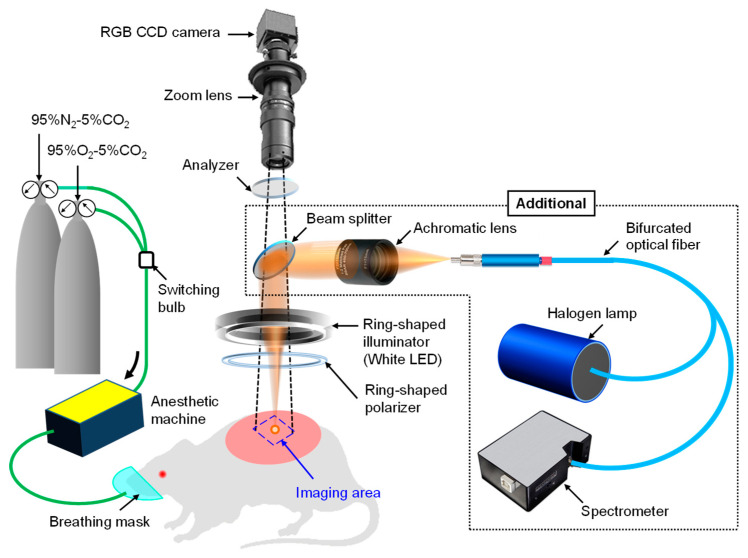
Schematic diagram of experimental configuration for image acquisitions and the spectral reflectance measurements.

Figure 11 shows a schematic illustration of the experimental system used in this study. A white light emitting diode (LED) (LA-HDF158A, Hayashi Watch Works Co., Ltd., Tokyo, Japan) illuminated the skin surface via a light guide and a ring-shaped illuminator with a polarizer. Diffusely reflected light was received by a 24-bit RGB CCD camera (DFK-21BF618.H, Imaging Source LLC, Charlotte, NC, USA) with a zoom lens and an analyzer to acquire an RGB image of 640 × 480 pixels. The primary polarization plate (ring-shaped polarizer) and the secondary polarization plate (analyzer) were set to be in a crossed Nicols alignment in order to reduce specular reflection from the skin surface. A standard white diffuser with 99% reflectance (SRS-99-020; Labsphere, North Sutton, NH, USA) was used to regulate the camera white balance. To evaluate the accuracy of the WEM, the reflectance spectra of skin were simultaneously measured by a fiber-coupled spectrometer (USB4000-XRS-ES, Ocean Optics Inc., Dunedin, FL, USA) with an integration time of 80 ms as reference data. Before the sequential measurements of RGB images and reflectance spectra, the area measured by the spectrometer was confirmed by projecting light from a halogen lamp (HL-2000, Ocean Optics Inc., Dunedin, FL, USA) onto the skin surface via one lead of a bifurcated fiber, lens, and beam splitter. The RGB image of the skin surface including the spot of light illuminated by the halogen lamp was stored on the PC, and then the size and coordinates of the spot were specified as the area measured by the spectrometer. After the halogen lamp was turned off, sequential measurements of RGB images and reflectance spectra were simultaneously performed. The region of interest (ROI) on the image of the skin surface was selected to be the same as the area measured by the spectrometer. Using the WEM, reflectance images ranging from 420 to 700 nm at intervals of 10 nm were reconstructed from the RGB image acquired at 30 ms of exposure time. This means that spectral images at 29 wavelengths can be obtained with a temporal resolution of 15 fps. Images at 16 wavelengths (500 to 650 nm at intervals of 10 nm) were then used to estimate the *C*_metHb_, *C*_HbO_, *C*_HbR_, and *C*_m_ images according to the above process. 

### 4.4. Animal Experiments 

Male Wister rats (*n* = 7) weighing 470 to 640 g were used for the animal experiments. All experimental procedures were conducted according to the protocols approved by the Animal Care Committee of Tokyo University of Agriculture and Technology (approval number 31–25, 4 June 2019 and approval number 31–75, 10 February 2020). The anesthesia of rats was performed with isoflurane, and maintained at a depth such that the rat had no response to toe pinching. After the induction of anesthesia, the dorsal region was shaved, and a depilatory agent containing thioglycolic acid was applied to the rat dorsal skin. 

First, we performed the measurements with four rats during methemoglobinemia. Rats were administered NaNO_2_ intraperitoneally at two different dose conditions of 37.5 and 50 mg/kg body weight (2-mL dosing volume) to induce methemoglobinemia. In the time series measurements during methemoglobinemia, both RGB images and diffuse reflectance spectra were acquired at 60 s intervals for 360 min. Second, we carried out measurements with three rats while varying the fraction of inspired oxygen (FiO_2_). The value of FiO_2_ was regulated by mixing 95%O_2_–5%CO_2_ gas and 95%N_2_–5%CO_2_ gas in an arbitrary ratio. Hyperoxia (FiO_2_ = 40%) was induced by 95%O_2_–5%CO_2_ gas inhalation, for which a breath mask was used under spontaneous respiration, whereas anoxia (FiO_2_ = 0%) was induced by 95%N_2_–5%CO_2_ gas inhalation. In the time series measurements, while varying FiO_2_, both RGB images and diffuse reflectance spectra were acquired at 10 s intervals for 35 min. Simultaneously, with the optical imaging for skin tissue, percutaneous arterial oxygen saturation (*SpO*_2_) and pulse distention (*PD*) were measured by a pulse oximeter (MOUSEOX Pulse Oximeter; Star Life Science, Oakmont, PA, USA) as systemic physiological parameters. The amplitude of the light absorption signal, due to the cardiac pulse, which corresponded to the change in the distention of the arterial blood vessels at the sensor location, was taken to be the *PD*. This can be used as an indicator of local blood flow or peripheral blood volume.

To evaluate the magnitude of the signal *M,* induced during methemoglobinemia and during changes in FiO_2_, we calculated the change in the signal based on the time series data. The signal before the administration of NaNO_2_ or at normoxia was selected as the control *M*_c_, which was subtracted from each of the subsequent signals M in the series. Each subtracted value, which demonstrated the change in the signal, *M* − *M*_c_, over time, was normalized by dividing by *M*_c_. The change in the signal is expressed as Δ*M* = {(*M* − *M*_c_)/*M*_c_}. The above calculation was applied to the time series of *C*_metHb_, *C*_HbO_, *C*_HbR_, *C*_HbT_, *StO*_2_, *C*_metHb_, *C*_m_, *SpO*_2_ and *PD*. 

### 4.5. Statistical Considerations 

We used only one area of each rat for data analysis of spectral images. The region of interest (ROI) of 300 × 300 pixels was set in each image and the mean and the standard deviation (SD) over the ROI were calculated for the analysis of time courses in *C*_metHb_, *C*_HbO_, *C*_HbR_, *C*_HbT_, *StO*_2_, and *C*_m_. Therefore, data are expressed as mean ± SD. To test the accuracy of the spectral reconstruction, the spectrum estimated by the WEM is compared with the spectrum measured by the spectrometer using a goodness-of-fit coefficient (GFC) [59]. The GFC is based on the inequality of Schwartz and is described as:

(21) $$\mathrm{GFC}=\frac{\left|\sum_{j}r_{mes}\left(\lambda_{j}\right)r_{est}\left(\lambda_{j}\right)\right|}{\sqrt{\left|\sum_{j}{\left[r_{mes}\left(\lambda_{j}\right)\right]}^{2}\right|}\sqrt{\left|\sum_{j}{\left[r_{est}\left(\lambda_{j}\right)\right]}^{2}\right|}}$$

where $r_{mes}\left(\lambda_{j}\right)$ is the measured original spectral data at the wavelength $\lambda_{j}$ and $r_{est}\left(\lambda_{j}\right)$ is the estimated spectral data at the wavelength $\lambda_{j}$. Hernández-Andrés et al. [61] suggested that colorimetrically accurate $r_{mes}\left(\lambda_{j}\right)$ requires a GFC > 0.995; a “good” spectral fit requires a GFC ≥ 0.999, and GFC ≥ 0.9999 is necessary for an “excellent” spectral fit.

## 5. Conclusions

In summary, the present study demonstrated the usefulness of a new non-contact imaging method based on spectral reflectance images reconstructed from a single snapshot of an RGB image by WEM for the simultaneous measurements of the percutaneous volume concentrations of methemoglobin, oxygenated hemoglobin, deoxygenated hemoglobin, and tissue oxygen saturation. In vivo experiments with rat dorsal skin before and after the administration of NaNO_2_ at doses of 37.5 and 50 mg/kg were performed to induce methemoglobinemia. The methemoglobin concentration rapidly increased with a half-maximum time of less than 20 min, reaching a maximal value nearly 60 min after the administration of NaNO_2_. The time required for returning to the normal levels increased proportionally with the dose, which implied that the methemoglobinemia was temporary. The values of tissue oxygen saturation significantly dropped after the administration of NaNO_2_, and then gradually increased for each dose condition, indicating temporary hypoxemia caused by methemoglobinemia. Time courses of total hemoglobin concentration and tissue oxygen saturation, while changing the fraction of inspired oxygen, coincided with well-known physiological responses to hyperoxia, normoxia, and hypoxia, which demonstrates the feasibility of using this method for monitoring changes in skin hemodynamics due to loss of tissue viability and vitality. The great advantages of this method are its simplicity and applicability, because the only devices required are a digital RGB image sensor with a known color profile, a white light source, and a personal computer.

## Figures and Tables

**Table 1:** Goodness-of-fit coefficient (GFC) obtained from four rats during methemoglobinemia.

Goodness-of-Fit Coefficient (GFC)						
Rat	Mean	±SD	Max	Min	Number of Spectra	Accuracy
#1	0.9990	0.0006	0.9999	0.9972	339	Good
#2	0.9996	0.0002	0.9999	0.9998	359	Good
#3	0.9999	0.0001	0.9999	0.9995	363	Excellent
#4	0.9993	0.0003	0.9999	0.9982	358	Good

**Table 2:** Goodness-of-fit coefficient (GFC) obtained from three rats during changes in FiO_2_.

Goodness-of-Fit Coefficient (GFC)						
Rat	Mean	±SD	Max	Min	Number of Spectra	Accuracy
#5	0.9991	0.0002	0.9996	0.9984	191	Good
#6	0.9983	0.0003	0.9990	0.9976	191	Colorimetrically accurate
#7	0.9981	0.0004	0.9992	0.9969	191	Colorimetrically accurate
